# The insights of Let‐7 miRNAs in oncogenesis and stem cell potency

**DOI:** 10.1111/jcmm.12861

**Published:** 2016-04-21

**Authors:** Xin Sun, Jian Liu, Chongwen Xu, Shou‐Ching Tang, Hong Ren

**Affiliations:** ^1^ Department of Thoracic Surgery and OncologyThe First Affiliated Hospital of Xi'an Jiaotong UniversityXi'anShaanxi ProvinceChina; ^2^ Department of OtorhinolaryngologyThe First Affiliated Hospital of Xi'an Jiaotong UniversityXi'anShaanxi ProvinceChina; ^3^ Georgia Regents University Cancer CenterAugustaGAUSA; ^4^ Tianjin Medical University Cancer Institute and HospitalTianjinChina

**Keywords:** Let‐7 miRNAs, regulatory loops, non‐coding RNAs, cancer stem cells, DNA methylation, anti‐cancer research, clinical application

## Abstract

The ability of the classic tumour‐suppressive let‐7 family to inhibit carcinogenesis, tumour progression, recurrence and pluripotency of cancer stem cells has generated significant interest in the field of cancer research. Through suppressing and degrading downstream‐targeted mRNAs, let‐7 affected most aspects of cell biology. It is perplexing how let‐7 affects oncogenesis, as the large influx of new miRNAs and other kinds of non‐coding RNAs are continuously defined. In this review, we delineate the complex functions of let‐7 and discuss the future direction of let‐7 research.

The discovery of regulatory non‐coding RNAs (ncRNAs) suggested the continuous evolving RNA in organisms, alongside proteins and DNA 1. Non‐coding RNAs can be grouped into the following subsets: long non‐coding RNAs (long ncRNAs, lncRNA), microRNAs (miRNAs), circular RNAs, short interfering RNAs (siRNAs), Piwi‐interacting RNAs (piRNAs), small nucleolar RNAs (snoRNAs), and other short RNAs 2, 3, 4. The extent of studies on non‐coding sequence transcription was recognized to be much greater than those coding sequences, most of which have been overlooked for years.

The siRNAs and/or miRNAs, including the let‐7 family of miRNAs, once dominated scientific journals. Renewed interest has been generated recently on the function of let‐7 in oncogenesis and in stem cell potency. An increasing number of reports have focused on long transcripts that act functionally as RNAs instead of let‐7. Enthusiasm for ncRNAs has shifted from miRNAs to lncRNAs, which are involved in chromatin remodelling, being involved in intricate regulatory nets 5, 6, 7. There are other aspects of let‐7 that are still worthy of further exploration, and we address the functions and mechanisms of let‐7 involved in cell and cancer biology in this review. To better understand regulatory mechanisms of let‐7, we also focus on let‐7 miRNA regulatory loops and the roles they play in inhibiting the self‐renewal of cancer stem cells (CSCs).

## Introduction of let‐7 miRNAs

The let‐7 family of miRNAs (Let‐7a/b/c/d/e/f/g/i and miR‐98) shares a common sequence, nucleotides 2 through 8 of their 5′ ends, which is important for target recognition 8. Let‐7 miRNAs were originally discovered to play crucial roles in the temporal regulation of *Caenorhabditis elegans*. Decreased let‐7 expression resulted in over‐proliferation and lack of terminal differentiation. Since then, let‐7 was shown to play important roles in the maintenance of normal differentiation and development of living organisms 9, 10.

The evidence for the influence of let‐7 in cancer came from early research that overexpression of let‐7 inhibited cancer cell proliferation. Subsequent studies of the targeted genes of let‐7 paved the way for a more complete understanding. Increased let‐7 can block tumour formation, progression and metastasis and can induce cell apoptosis through targeting and degrading downstream oncogenes through imperfect complementary binding to the 3′UTR of mRNAs (post‐transcriptional regulation) 11. Let‐7 can also regulate self‐renewal, differentiation, and the recently defined cell polarity of CSCs, which are regarded as the putative root for cancer progression, heterogeneity and therapy resistance. Forced let‐7 expression inhibited mammosphere formation and the carcinogenesis of stem cells, and reduced the proportion of undifferentiated cells *in vitro via* different pathways; similarly, antagonizing let‐7 miRNAs by anti‐sense oligonucleotides enhanced self‐renewal and prompted aggressiveness 12. Let‐7 miRNAs may also function as clinical indicators for prognosis analysis and treatment evaluation in breast cancer. Numerous oncogenes and signalling pathways were demonstrated to be targets of let‐7 miRNAs: Ras, HMGA2, cyclin d1/2/3, cyclin A, CDK4/6, c‐Myc, DICER1, Lin28, *etc*. 6, 8.

## Roles in anticancer research and treatment

### The application of let‐7 in clinical application

As more molecular mechanisms were identified, the interest in let‐7 miRNAs extended to clinical medicine. In fields of clinical practice, let‐7 miRNAs could be used to classify tumours and predict prognosis 12. The regulation of let‐7c was confirmed to be correlated with oestrogen and progesterone receptor status, and overexpressed let‐7c blocks oestrogen activation of Wnt activity. Let‐7a/f was associated with lymph node metastasis, and let‐7c/d was analogous with a high proliferation index in breast cancers 13. Let‐7a/g was also shown to be correlated with lung cancer survival 14. In anticancer research, let‐7 miRNAs are perfectly capable of killing tumour cells or at least significantly inhibiting cell biological functions in lung cancer and breast cancer 15, 16, and more specifically, the injected exosomes delivered let‐7 miRNAs functioning by targeting EGFR into the EGFR‐expressing breast cancer cells in RAG2^−/−^ mice showed a platform for miRNA replacement therapies 17. Nanoparticle‐based let‐7 replacement therapy was successfully applied *in vivo*. TUTase‐mediated Lin28, which is a facile target inhibited by pharmacological compounds, can relieve let‐7 maturation repression and shows promising value in clinical practice 18, 19. The systemic delivery of synthetic let‐7b mimics, in complex, neutral lipid emulsion, into mice with tumour shows a decreased tumour burden in mouse models 20.

### The uncertainty of let‐7 functions

Let‐7 may not be that effective. Let‐7 results showed a repressive function on tumour‐suppressive caspase‐3 and BCL2‐associated X protein gene (BAX) in recent studies (acting as tumorigenesis factor) 16, 21. The tumour suppressor p53 showed inhibitory effects on let‐7 expression in anticancer radiotherapy 22, 23, 24. The roles of let‐7 in cancer still appear to be unresolved, and our studies revealed that oncogenic cyclin D1 and oestrogen activation both induced certain let‐7 expression, indicating the complicated roles of let‐7 in the regulatory nets. Another group also found that let‐7 miRNAs could be selectively secreted in metastatic gastric cancer cell lines 15. These emerging oncogenic functions of the traditionally regarded tumour suppressor let‐7 require more exploration. The comprehensive understanding of let‐7 in regulatory loops, signal pathways and cancer cell subtypes will make let‐7 or other miRNA‐based treatments more precise and yield therapeutic advances for anti‐cancer treatment.

## Critical focus area

**Figure 1:**
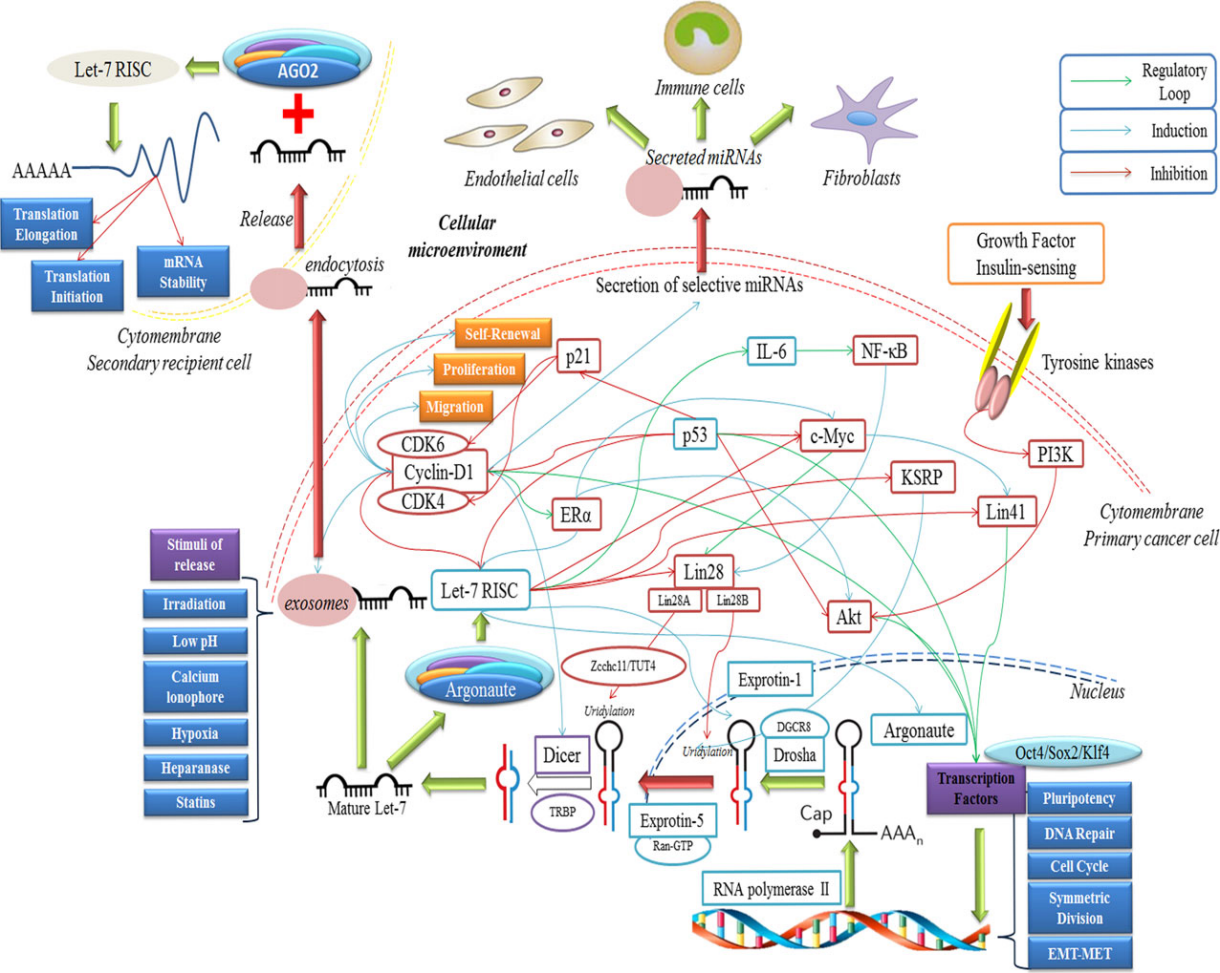
The regulatory functions and mechanisms of let‐7 in cell biology. Let‐7 is involved in multiple intracellular and extracellular functions through targeting and degrading the 3′UTR of certain mRNAs. Moreover, the feedback regulatory loops between let‐7 and its targeted genes constitute a more complicated and interesting network, centering on let‐7. Intracellular let‐7 may be also secreted to affect adjacent cells, exerting multiple effects.

Let‐7 functions in normal cell differentiation, fate determination and especially in cancer development and in CSCs. How let‐7 regulates CSCs to eradicate the tumour bulk is important. Its influence on transcription factors could regulate the expression of multiple genes. Let‐7 may be involved in not only regulatory loops between downstream genes and itself but it also may be involved in the extensive cellular function modifications (partially showed in Fig. 1). Here, we focus on other fields that were neglected.

### Let‐7 involvement in regulating CSCs

Human tumours are heterogeneous groups, containing slowly proliferating CSCs that resist common chemoradiotherapy 6, 25. This term means that cancer could arise from a few cells that have the capacity to generate the numerous cells with different types, contributing to tumour recurrence 26. When surgeons remove primary tumours, with perfect marginal clearance, the CSCs do not necessarily go away 27. Despite advances in cancer diagnosis and treatments, many patients still have poor prognosis with reduced overall survival 28. MiRNAs have been shown to play crucial roles in sustaining CSC identity through regulating multiple networks of transcription factors and RNA‐binding proteins, among which let‐7 was identified to control cell proliferation, self‐renewal differentiation and cell pluripotency 6, 29.

Effects on cell differentiation and polarity aligned let‐7 with cancer growth and CSC characteristics. The let‐7 miRNA family was proven to inhibit the cellular reprogramming process. Reprogramming well‐differentiated cells into induced pluripotent stem cells had a great significance on tissue repair and tumour occurrence. The loss of let‐7 in CSCs resulted in more aggressive features of self‐renewal and chemoradiotherapy resistance 6, 30. p53 takes part in affecting embryonic and CSC reprogramming by repressing Nanog, Sox2, Oct4, Klf4 and c‐Myc. However, p53 has also demonstrated the ability to suppress let‐7 expression and formed regulatory loops in regulating epithelial mesenchymal transformation and DNA damage response. Therefore, the understanding of the relationships between p53 and let‐7 will have great benefits on cell pluripotency and the generation or maintenance of CSCs. Worringer *et al*. recently confirmed that through suppressing the Lin41/EGR1 pathway, let‐7 could inhibit reprogramming efficiently 31. They also established that through Lin41, let‐7 inhibition enhanced Oct4/Sox2/Klf4‐mediated cell reprogramming; let‐7 inhibition together with Oct4/Sox2/Klf4 showed a high level of reprogramming efficiency compared to Oct4/Sox2/Klf4/c‐Myc 32. Lin28 promoted reprogramming by down‐regulating let‐7, and with the help of Oct4, Sox2 and Nanog, Lin28 is sufficient to reprogramme human somatic cells to pluripotent stem cells, or possibly, CSCs 33, 34.

**Figure 2:**
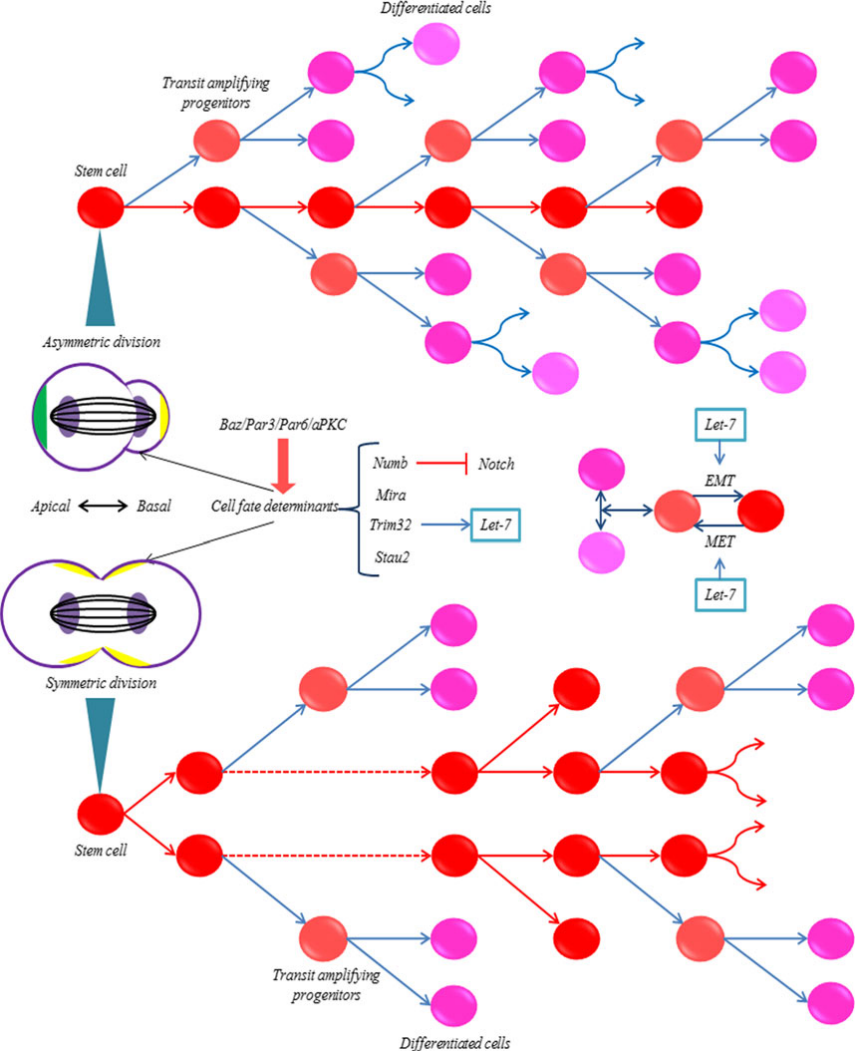
The regulations of self‐renewal and let‐7 involved stem cell generation. Asymmetric division type helps maintain a balance of primary stem cells and differentiated cells, resulting in two unequal daughter cells, one of which resembles the parent stem cell, with another differentiating into distinct cell types. Cell determinants were segregated to the cytoplasm of one daughter cell differently, associated with membranes, and centrosome of other constituents. Symmetric cell division types made stem cells divide symmetrically, producing two stem cell daughters and expanding the stem cell numbers. The stem cells transmit amplifying progenitors, and well differentiated cells could be reprogrammed when they are triggered by cytokines, signals, transcription factors and micro‐environmental niches. Let‐7 could function in influencing some key factors related to division types.

**Figure 3:**
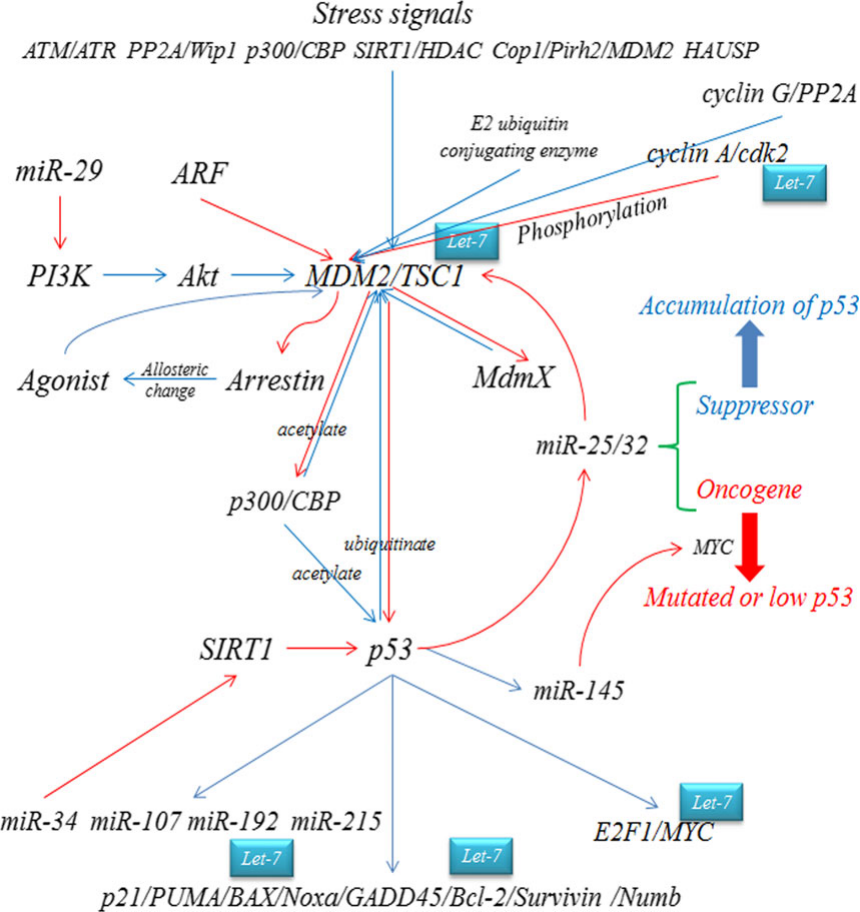
TP53 dominated cell polarity direction. P53 was used as an example to illustrate the functions of genes and signalling pathways in stem cell division, which is regulated mainly by E3 ubiquitin ligase, functioning in Notch signalling. As post‐transcriptional regulators, let‐7 and many miRNAs could probably make similar functions through regulating mRNAs implicated in this process, with specific roles determined in different situations.

Failing to undergo asymmetric cell division helped CSCs to generate two undifferentiated daughter cells, dividing symmetrically and resulted in more uniform stem cells 35. The symmetric division produced two stem cells capable of self‐renewal and forming the tumour (Fig. 2) 36, 37. Mutations or loss of crucial suppressive regulators that perturb cell polarity can cause neoplastic emergence through losing control of CSC over‐proliferation, such as Notch, Wnt and TGF‐β signalling. Also, several key factors, such as p53, p63, numb, Akt and TSC1/2, were reported to be related to the polarity of stem cells, and our ongoing studies suggested let‐7 may be involved in these regulations through interacting with different parts, such as Let‐7/ESR1/Wnt, and Let‐7/Akt/Hedgehog (Fig. 3) 25, 38, 39.

### Secreted let‐7 through exosomes

Cells continuously release soluble factors and deposit membrane‐derived vesicles into the extracellular microenvironment, such as mast cells, dendritic cells, B‐lymphocytes platelets, neurons, adipocytes, endothelial cells and epithelial cells 7. The smaller class of nanometer‐sized (30–200 nm) vesicles is called exosomes. They are membrane‐derived vesicles, acting as important mediators of intercellular communication to recipient cells. For example, exosomes from T cells, B cells and dendritic immune cells function by communicating with antigen‐presenting cells. In malignancies, this process entails the transfer of oncogenic signals to surrounding cells *via* the microenvironment, or maybe into the body circulation. Exosomes were first observed in reticulocyte maturation, functioning as cellular garbage disposals 40. Exosomes were identified as important mediators of cellular communication, involved in both normal physiological processes and disease progression. However, this regulation only applies to the selected miRNAs.

It is well agreed that carcinogenesis is dependent on a relationship between cancer cells and the stromal microenvironment. Tumour‐derived exosomes are generally considered pro‐tumorigenic, possessing many tumorigenic functions to transfer their information to recipient cells and to promote cancer‐stimulatory activities 7. Various tumour cells have been shown to secrete exosomes more often than adjacent normal cells, allowing the transfer of tumour‐associated lipids, proteins, mRNAs and especially, miRNAs. The exosomes mediate intercellular communication based on miRNAs that regulate target gene expression will help to understand the basic biology of cancer progression and the development of therapeutic approaches.

Cyclin D1 could increase pre‐let‐7a, pre‐miR‐16 and pre‐miR‐17/20 through inducing DICER1, and also was correlated with mature let‐7b (Fig. 1) 6, 41, 42, 43. They also identified a bulk of endogenous miRNAs that could be selectively secreted into the cellular matrix in cyclin D1^−/−^ cells, which may have a direct feedback loop with cyclin D1 or the epigenetic DICER1 (Fig. 1) 41. Let‐7 may be preferentially secreted, however, the mechanisms for selective packaging and release of let‐7 are barely known. Let‐7 interacted with the recipient cells, and the role let‐7 played in the microenvironment is still unclear. To promote or inhibit nearby cancer cells, stem cells and CSCs still need further exploration.

### Newly defined regulating axis and regulatory loops

The newly identified regulatory loops between genes and let‐7 were confirmed to control the fate of cancer cells. This allowed let‐7 miRNAs to cooperate with multiple transcription factors or directly regulate the targeted gene expression. This extended the repertoire function of familiar let‐7 and its numerous targeted genes 41. Previously we discussed the let‐7 expression may actually be influenced by cyclin D1 expression, and let‐7 also may decrease the ERα expression 11, 41, 43. With the existing regulatory loop between ERα and cyclin D1, the let‐7/cyclin D1/ERα loop may dominate in the ERα signalling in mammary glands *in vivo* and breast cancers *in vitro* 44, 45.

Lin28 is an RNA‐binding protein that was first described in a *C. elegans* screen for heterochronic genes, which regulate developmental timing and growth, and has recently been linked in mammals 46, 47. The loss of Lin28 caused precocious larval progression in adults, whereas the gain of Lin28 delays larval progression 48, acting as a pluripotency factor in the control of cellular stemness and early embryonic development 1. Mammalian Lin28 exists with two highly conserved paralogues, Lin28a and Lin28b, both repressing let‐7; the inverse relationship with let‐7 expression was noticed in *C. elegans* along with the discovery of the let‐7 miRNAs family. Lin28a is expressed in HER2‐positive breast cancer cells, whereas Lin28b expression characterizes triple‐negative breast cancer 49. Lin28a recruits the 3′ terminal uridylyl transferase Zcchc11 or Zcchc6i in the cytoplasm, adding an oligouridine tail to pre‐let‐7, and uridylated pre‐let‐7 is refractory to be processed by Dicer, while Lin28b could repress ler‐7 processing through the Zcchc11‐independent mechanism in the nucleus 49, 50, 51, 52. Lin28 blocks the precursor processing of let‐7, finally decreases let‐7 biogenesis and maturation. Chang *et al*. of Boston Children's Hospital recently identified the exonuclease Dis3l2 was responsible for the decay of uridylated per‐let‐7 *in vitro* and *in vivo*, establishing the 3′ oligouridylation of let‐7 as an RNA decay signal for Dis3l2, and a block to Dicer processing 55. These results inserted Dis3l2 into the Lin28‐let‐7 loop as a new component, which was essential for decay of uridylated pre‐let‐7 50. In germ cell tumours, Lin28 depletion relieves let‐7 expression, and the restored let‐7 effectively repressed downstream oncogenes, such as MYCN, CCNF, RRM2, MK167 and AURKB; meanwhile, the team discovered that let‐7e could also reduce Lin28 51, 53. In another study, Lin28a reactivation improved hair regrowth by promoting anagen in hair follicles and accelerated regrowth of cartilage, bone and mesenchyme after ear and digit injuries by inhibiting let‐7 microRNA biogenesis 48.

C‐Myc can regulate cell proliferation, apoptosis and irregular c‐Myc, which is one of the most frequently observed abnormalities in human malignancies. As one of the targeted genes of let‐7 miRNAs, c‐Myc can also bind to the promoters of let‐7a, let‐7d, let‐7f and let‐7g pri‐miRNAs, decreasing their expression 21. Given that Lin28 is up‐regulated by c‐Myc, it can also stimulate Lin28 expression by directly binding to its promoter. The repressed let‐7 may be ascribed to c‐Myc or Lin28 expression partly 19, which extended the Lin28/let‐7 regulatory loop 9, 54. Take into consideration that the positive feedback loop may exist between NF‐κB and interleukin (IL)‐6, let‐7 and IL‐6, and the loop of IL‐6/NF‐κB/Lin28/let‐7/IL‐6. Together, the research of Lin28/let‐7 axis established a double‐negative feedback loop, ranging from embryonic development, oncogenesis, embryonic stem cells (ESCs), to CSCs.

Tissue homoeostasis and repair needs assistance from stem cells, but this may be harmful by inducing malignancy in individuals. Ageing reduced tissue maintenance, and regeneration may result in a concomitant loss of stem cells, with decreased expression of insulin‐like growth factor (IGF)‐II messenger RNA‐binding protein (Imp), and a conserved family of RNA‐binding proteins 43. One group demonstrated that let‐7 increases in ageing/differentiated cells, and then the mature let‐7 decreased Imp, destabilizing the self‐renewal factor Unpaired (Upd) mRNA and finally establishing the let‐7/Imp/Upd/JAK/STAT signalling axis in a stem cell niche 43. Since differentiation and regeneration contradict each other, the harmful Lin‐28/let‐7 and let‐7/Imp axis in tissue repair and pluripotency may be of greater benefit in promoting CSC differentiation (decrease the ‘stemness’).

Alpha glycoprotein subunit (αGSU), common to three pituitary hormones, is unstable and its half‐life increases during pituitary development. Let‐7b/c could degrade the RNA‐binding protein, KSRP mRNA, by directly binding to the 3′UTR, which favours the biogenesis of let‐7 miRNAs family members. This established a negative feedback loop that KSRP induces the maturation of let‐7b/c, and in turn, post‐transcriptionally suppresses KSRP itself, leading to the stabilization of αGSU mRNA, which is increased by let7b/c and decreased by KSRP 56. The research conducted by Dimitrios and his team indicated the crucial auto‐regulation of let‐7 biogenesis, which formed let‐7 and Argonaute 22. They demonstrated that the Argonaute protein in *C. elegans* (ALG‐1) can bind to let‐7 miRNA primary transcripts through the 3′ sites and promote let‐7 maturation. This process is also mediated by mature let‐7 miRNA, creating a positive‐feedback loop in nuclear fractions.

### Interactions with ncRNAs

It is accepted that miRNAs regulate mRNAs in the cytoplasm of original cells or adjacent cells, as discussed above. Furthermore, there may exist a relationship between miRNAs and ncRNAs, with the identifications of the proteins transporting miRNAs from the cytoplasm into the nucleus, which could be controlled by non‐coding RNAs 57. With the help of exporting‐1, mature let‐7 can be shuttled through the nuclear‐cytoplasmic gate in a complex containing Argonaute (Ago) protein, which is the key component in forming RNA‐induced silence complex 58. MiR‐671 and miR‐709 can regulate miRNAs and lncRNAs respectively 57, 59. It is interesting that mature let‐7, with the help of Ago proteins can bind to and promote the processing of its own primary transcript, forming a direct auto‐regulatory loop 57. However, whether nuclear let‐7 miRNAs can regulate other ncRNAs is still being explored.

LncRNAs can bind to and regulate miRNA functions, and a large‐scale regulatory network of lncRNAs and miRNAs is still being explored 60. The research conducted at the Yale Stem Cell Center indicates the possible relationship between miRNAs and lncRNAs. In their research, let‐7 was demonstrated to be modulated by H19, a long non‐coding RNA that plays crucial roles in growth control, human genetic disorders and cancer, belonging to a highly conserved imprinted gene cluster, which could control various physiological and pathological processes through regulating let‐7 family members 61. Previously, miR‐675 was confirmed to be produced from H19's first exon through Drosha processing 62.

Latest studies revealed the naturally occurring Circular RNAs (CircRNAs), forming a covalently closed continuous loop, which belong to a family of ncRNAs. CircRNAs can function as miRNAs sponges, regulators of splicing and transcription 63. CircRNA for miRNA‐7 (ciRS‐7) acts as an anti‐complementary miRNA ‘sponge’ to adsorb and quench miRNA‐7, and deficits in ciRS‐7 were expected to increase ambient miRNA‐7 64. Together with other findings of circRNAs, the deficits in miRNA sponging process and ambient up‐regulation of specific inducible miRNAs may function *via* down‐regulation of gene expression characteristic of tumour initiation and progression. However, the let‐7‐specific circRNA has not been confirmed for now.

### Let‐7 interactions with DNA methylation

**Figure 4:**
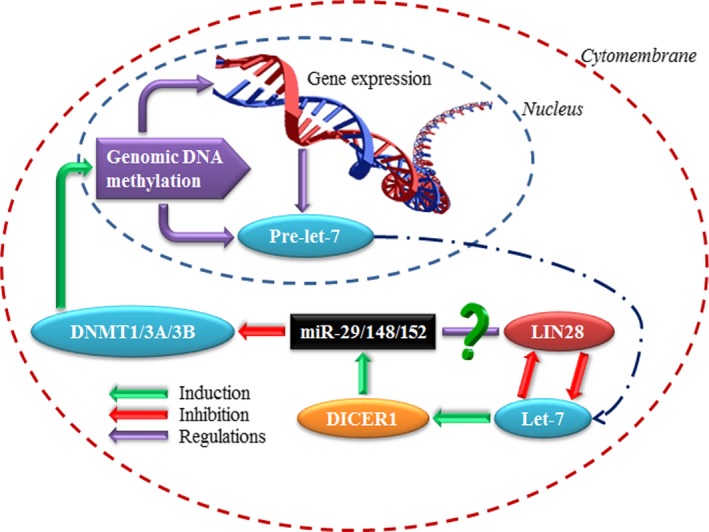
Methylation–miRNA regulatory loop. EpimiRNAs regulate whole gene expression patterns and some methylation‐associated regulators, such as DNMTs and TETs, by which epimiRNAs can regulate genomic DNA methylation levels. CpG island methylation status can regulate gene expression containing some miRNAs, such as let‐7a‐3, which can regulate other miRNA family members through DICER.

Accumulating studies have shown that one‐third of all human miRNAs has a CpG island in the upstream region and may be regulated by DNA methylation which might contribute to human tumorigenesis 65. We summarized the findings regarding some of the more intensively studied miRNAs for which expression is regulated by epigenetic mechanisms (Fig. S1). On the other hand, some microRNAs (so‐called ‘epi‐miRNAs’) may even target the epigenetic machinery itself, such as the DNA methyltransferases (DNMTs) and their antagonist retinoblastoma‐like 2 (Fig. 4) 66. For example, miR‐148a and miR‐152 target DNMT3B and DNMT1, respectively, and regulate genomic DNA methylation, as were illustrated in Figure 4 67, 68. Mouse models have shown that miRNAs are able to regulate ten‐eleven‐translocation protein (TET) gene expression by targeting their 3′UTR and then participate in active DNA demethylation 69, 70. Recent studies showed that miR‐22 was responsible for the down‐regulation of all three TET genes, and its conditional expression in a transgenic mouse model led to reduced levels of 5‐hmC and development of hematopoietic malignancies 71.

The let‐7a‐3 gene was hypermethylated in normal tissues of placenta, brain, bone marrow, colon, skin and lung tissues, but hypomethylated in some lung cancer samples. Methylation level of let‐7a‐3 correlates inversely with let‐7a‐3 expression levels. DNMT1 and DNMT3B double‐knockout HCT116 colon cancer cells showed that let‐7a‐3 methylation is cooperatively maintained by the DNA methyltransferases DNMT1 and DNMT3B just like other genomic regions 72. In epithelial ovarian cancer, let‐7a‐3 methylation slightly affected the expression of let‐7a‐3, and then let‐7a‐3 may regulate IGF‐II *via* targeting of IGF2‐binding proteins (IMP‐1/2). In addition, patients with methylated let‐7a‐3 seemed to have reduced risk for death compared with those without 73.

### The value in clinical application

Circulating miRNAs *via* exosomes or circulating tumour cells can be used as a diagnostic marker, particularly for asymptomatic patients 74, 75, 76, which could be viewed as a promising strategy in the early diagnoses of patients with malignancies or precancerous lesions. Several studies have examined the miRNA profile from the plasma of patients by isolating exosomes, however, considering that the expression levels of miRNAs may differ from primary tumour tissues and plasma, the roles of certain miRNA and the variation tendency should be reappraised besides the original findings. In *in vitro* studies, let‐7 expression was detected from exosomes of mast cells, metastatic gastric cells and melanoma cells, revealing that let‐7 miRNAs were enriched in exosomes, indicating the detections of let‐7 in plasma could be achieved by the isolation of exosomes. Secreted miRNA profiles have been tentatively applied in clinical practice, and recently were found to be carriers of genetic information, bringing new insight to research with the help of natural nanoparticles: not only as detection indexes but also as therapeutic weapons.

Chemically modified miRNA analogues were applied to demonstrate the functional activities based on regulated miRNA functions in many types of diseases, especially cancer. The soluble oligonucleotides are suitable for injection, intravenous or subcutaneous; and these kinds of delivery methods were successfully used in most cases 77. Upon uptake, certain miRNA analogues could bind to target mRNAs suppressing mRNAs functions. The miRNA sponge was also accepted as an innovative concept to regulate multiple miRNAs 14. The sponge could produce a single segment of RNAs consisting of multiple repeats of tandem‐binding sites, complementary to seed regions of certain miRNAs 78. Through base pair‐dependent interaction to the seed region in the miRNAs, the sponge leads to a reduction in active miRNAs. Adenoviral and lentiviral constructs of miRNA sponges utilize high cytoplasmic expression through optimization of the sponge export from nuclease, allowing chronic inhibition of miRNAs 79. The interactions between let‐7 and H19 lncRNA was exactly the molecular sponge pattern 62. LncRNA CCAT1 promotes carcinoma progression by acting as a let‐7 sponge, suggesting the LncRNAs controlled let‐7 are applicable 80, 81. In conclusion, let‐7 may be a powerful tool to fight against cancer, and may become a candidate for cancer diagnosis and prognosis, acting as suitable agents for anti‐cancer therapy, and be applied by using miRNA sponge, adenoviral and lentiviral constructs, miRNA analogues, natural nanoparticles and soluble oligonucleotides.

## Conclusion and expectation

It is now accepted that genetic information could be transcribed into ncRNAs, which are mostly spliced and processed into smaller products. They comprise a hidden layer of genetic information, and control various gene expression levels in cellular physiology. They develop through regulating chromatin architecture, epigenetic transformation, transcription and RNA splicing and translation by determining the cell type, tissue homoeostasis and their potential involvement in gene transcription programmes 82, 83. Let‐7 has been known as one of the earliest and most classic miRNAs since miRNAs were found, playing critical roles in carcinogenesis as an unalterable suppressor. The entire set of let‐7 in malignant transformation and cancer progression are exploited to achieve novel modifications in the existing diagnostic and therapeutic approaches to cancer management 84, which will be systematically identified.

The cross‐reactions and auto‐regulatory loops were challenging the inherent modes that let‐7 and other miRNAs followed. Fine tuning of gene expression by let‐7 is known to have significant clinical and biological impacts, and further work on uncovering the mechanistic details, especially identifying novel regulators will help to understand how to treat and control human disease. The mono‐uridylation of let‐7 by Lin28 or TUTases dominates in the let‐7 biogenesis, which could influence Dicer processing and add a new research direction in let‐7 regulation nets 17. As to the secreted let‐7 miRNAs, most tumour‐derived exosomes were related to the immune system, and recently, studies have turned to focus on their pro‐tumorigenic functions of the transfer of the oncogenic miRNAs to recipient cells. Similarly, suppressive miRNAs secreted into the microenvironment could be taken up by recipient cells, exerting gene silencing and growth inhibition. Also, it has to be kept in mind that cytokine, chemokine and growth factors in the extracellular matrix (micro‐environmental niches) also play important roles in the development of heterogeneous tumours, sustaining the perpetuating CSCs 27.

## Conflict of interest

All co‐authors implicated in this research declare that they have no conflict of interest.

## Supporting information


**Figure S1** miRNAs signatures regulated by epigenetic mechanisms.
