# Supplementary material for: The insights of Let‐7 miRNAs in oncogenesis and stem cell potency
Source: J Cell Mol Med. 2016 Apr 21;20(9):1779–88. doi: 10.1111/jcmm.12861 (PMC4988292; doi:10.1111/jcmm.12861)
Supplement: Supplementary file 1 — Figure S1 miRNAs signatures regulated by epigenetic mechanisms. [file JCMM-20-1779-s001.doc]

Supplemental Figure 1 miRNAs signatures regulated by epigenetic mechanisms

| **miRNAs** | **Locus** | **Targets** | **Cancer types** | **Reference** |
| --- | --- | --- | --- | --- |
| **miR-1** | 20 | FoxP1/HDAC4/MET | Hepatocarcinoma |  |
| **miR-9-1** | 1 | FGF family/Claudin family | Breast, ovarian, pancreas, hematological, renal, colorectal cancer, gastric cancer |  |
| **miR-9-2** | 5q14.3 |  | gastric cancer |  |
| **miR-9-3** | 15q26.1 | E-cadherin | Colorectal, melanoma, head and neck, gastric cancer |  |
| **miR-10a** | 21q21.32 | HOXA3/ HOXD10 | Hematological, colon |  |
| **miR-34a** | 1p36.23 | CDK6/CD44/Notch1 | Hematological, prostate, breast, renal, colorectal, ovarian, lung, pancreatic, bladder, melanoma |  |
| **miR-34b/c** | 11q23.1 | CDK6/ E2F3/ CREB/MYC | Gastric, ovarian, lung, colon, melanoma, head and neck |  |
| **miR-107** | 10q23.31 | CDK6 | Pancreatic |  |
| **miR-124a** | 20q13.33 | CDK6/EBPa/VIM/SMYD3 | Colon, gastric, hematological, cervical, liver glioblastoma, breast |  |
| **miR-126** | 9q34.3 |  | Bladder, prostate |  |
| **miR-127** | 14q32.31 | BCL6 | Prostate, bladder |  |
| **miR-129-2** | 11p11.2 | SOX4 | Gastric, endometrial, colorectal |  |
| **miR-132/212** | 17p13.2 |  | Hematological |  |
| **miR-137** | 1p21.3 | CDK6/E2F6/ LSD-1/noca2 | Oral, colorectal, glioblastoma, |  |
| **miR-145** | 5 | OCT/SOX2/KLF4 | Prostate |  |
| **miR-148a** | 7p15.2 | TGIF2 | Colorectal, melanoma, head and neck, breast |  |
| **miR-152** | 17q21.32 |  | Breast |  |
| **miR-181a/b-2/c** | 9q33.3 | PLAG1 | Hematological |  |
| **miR-181c** | 19 | K-Ras/Notch4 | Gastric |  |
| **miR-193a** | 17q11.2 | E2F6/PTK2/MCL1 | Oral |  |
| **miR-196a-2** | 12q13.13 |  | Breast |  |
| **miR-196b** | 7p15.2 |  | Hematological |  |
| **miR-199a-1** | 19p13.2 | ERK2/MET | Lung, breast, prostate, oral |  |
| **miR-199a-3p** |  | mTOR, c-Met | Hepatocarcinoma |  |
| **miR-141/200c** | 12p13.31 | ZEB1/ZEB2 | Colorectal, breast, lung |  |
| **miR-200a/b/429** | 1p36.33 | ZEB1/ZEB2/ SOX2/ KLF4 | Colorectal, breast, lung |  |
| **miR-203** | 14q32.33 | ABL1/ BCR-ABL1/ Bmi-1 | Hematological, liver |  |
| **miR-205** | 1 | ZEB1/ZEB2 | Bladder |  |
| **miRNA-335** | 7 | SOX4/TNC | Breast |  |
| **miR-342** | 14q32.2 | PDGFRA/ RASA1 | Colorectal |  |
| **miR-370** | 14q32.31 | MAP3K8 | Cholangiocarcinoma |  |
| **miR-512-5p** | 19q13.41 | MCL-1 | Gastric |  |
| **miR-663** | 20p11.1 |  | breast |  |
| **let-7a-3** | 22q13.31 | IGF-II | Ovarian, lung, |  |

1. Datta, J., et al., *Methylation mediated silencing of MicroRNA-1 gene and its role in hepatocellular carcinogenesis.* (1538-7445 (Electronic)).

2. Roman-Gomez, J., et al., *Epigenetic regulation of microRNAs in acute lymphoblastic leukemia.* (1527-7755 (Electronic)).

3. Bandres, E., et al., *Epigenetic regulation of microRNA expression in colorectal cancer.* (1097-0215 (Electronic)).

4. Lujambio, A., et al., *A microRNA DNA methylation signature for human cancer metastasis.* (1091-6490 (Electronic)).

5. Laios, A., et al., *Potential role of miR-9 and miR-223 in recurrent ovarian cancer.* (1476-4598 (Electronic)).

6. Hildebrandt, M.A., et al., *Hsa-miR-9 methylation status is associated with cancer development and metastatic recurrence in patients with clear cell renal cell carcinoma.* (1476-5594 (Electronic)).

7. Tan, H.X., et al., *MicroRNA-9 reduces cell invasion and E-cadherin secretion in SK-Hep-1 cell.* (1559-131X (Electronic)).

8. Tsai, K.W., et al., *Aberrant hypermethylation of miR-9 genes in gastric cancer.* (1559-2308 (Electronic)).

9. Hsu, P.Y., et al., *Xenoestrogen-induced epigenetic repression of microRNA-9-3 in breast epithelial cells.* (1538-7445 (Electronic)).

10. Han, L., et al., *DNA methylation regulates MicroRNA expression.* (1555-8576 (Electronic)).

11. Lodygin, D., et al., *Inactivation of miR-34a by aberrant CpG methylation in multiple types of cancer.* (1551-4005 (Electronic)).

12. Toyota, M., et al., *Epigenetic silencing of microRNA-34b/c and B-cell translocation gene 4 is associated with CpG island methylation in colorectal cancer.* (1538-7445 (Electronic)).

13. Kozaki, K., et al., *Exploration of tumor-suppressive microRNAs silenced by DNA hypermethylation in oral cancer.* (1538-7445 (Electronic)).

14. Pigazzi, M., et al., *miR-34b targets cyclic AMP-responsive element binding protein in acute myeloid leukemia.* (1538-7445 (Electronic)).

15. Lee, K.H., et al., *Epigenetic silencing of MicroRNA miR-107 regulates cyclin-dependent kinase 6 expression in pancreatic cancer.* (1424-3911 (Electronic)).

16. Lehmann, U., et al., *Epigenetic inactivation of microRNA gene hsa-mir-9-1 in human breast cancer.* (0022-3417 (Print)).

17. Lujambio, A., et al., *Genetic unmasking of an epigenetically silenced microRNA in human cancer cells.* (0008-5472 (Print)).

18. Ando, T., et al., *DNA methylation of microRNA genes in gastric mucosae of gastric cancer patients: its possible involvement in the formation of epigenetic field defect.* (1097-0215 (Electronic)).

19. Furuta, M., et al., *miR-124 and miR-203 are epigenetically silenced tumor-suppressive microRNAs in hepatocellular carcinoma.* (1460-2180 (Electronic)).

20. Saito, Y., et al., *Epigenetic therapy upregulates the tumor suppressor microRNA-126 and its host gene EGFL7 in human cancer cells.* (1090-2104 (Electronic)).

21. Saito, Y., et al., *Specific activation of microRNA-127 with downregulation of the proto-oncogene BCL6 by chromatin-modifying drugs in human cancer cells.* (1535-6108 (Print)).

22. Suh, S.O., et al., *MicroRNA-145 is regulated by DNA methylation and p53 gene mutation in prostate cancer.* (1460-2180 (Electronic)).

23. Pallasch, C.P., et al., *miRNA deregulation by epigenetic silencing disrupts suppression of the oncogene PLAG1 in chronic lymphocytic leukemia.* (1528-0020 (Electronic)).

24. Hashimoto, Y., et al., *Involvement of epigenetically silenced microRNA-181c in gastric carcinogenesis.* (1460-2180 (Electronic)).

25. Hoffman, A.E., et al., *microRNA miR-196a-2 and breast cancer: a genetic and epigenetic association study and functional analysis.* (1538-7445 (Electronic)).

26. Kim, S., et al., *MicroRNA miR-199a* regulates the MET proto-oncogene and the downstream extracellular signal-regulated kinase 2 (ERK2).* (0021-9258 (Print)).

27. Fornari, F., et al., *MiR-199a-3p regulates mTOR and c-Met to influence the doxorubicin sensitivity of human hepatocarcinoma cells.* (1538-7445 (Electronic)).

28. Wellner, U., et al., *The EMT-activator ZEB1 promotes tumorigenicity by repressing stemness-inhibiting microRNAs.* (1476-4679 (Electronic)).

29. Vrba, L., et al., *Role for DNA methylation in the regulation of miR-200c and miR-141 expression in normal and cancer cells.* (1932-6203 (Electronic)).

30. Neves, R., et al., *Role of DNA methylation in miR-200c/141 cluster silencing in invasive breast cancer cells.* (1756-0500 (Electronic)).

31. Ceppi, P., et al., *Loss of miR-200c expression induces an aggressive, invasive, and chemoresistant phenotype in non-small cell lung cancer.* (1557-3125 (Electronic)).

32. Chen, J., et al., *Overexpression of miR-429 induces mesenchymal-to-epithelial transition (MET) in metastatic ovarian cancer cells.* (1095-6859 (Electronic)).

33. Park, S.M., et al., *The miR-200 family determines the epithelial phenotype of cancer cells by targeting the E-cadherin repressors ZEB1 and ZEB2.* (0890-9369 (Print)).

34. Wiklund, E.D., et al., *Coordinated epigenetic repression of the miR-200 family and miR-205 in invasive bladder cancer.* (1097-0215 (Electronic)).

35. Bueno, M.J., et al., *Genetic and epigenetic silencing of microRNA-203 enhances ABL1 and BCR-ABL1 oncogene expression.* (1878-3686 (Electronic)).

36. Tavazoie, S.F., et al., *Endogenous human microRNAs that suppress breast cancer metastasis.* (1476-4687 (Electronic)).

37. Grady, W.M., et al., *Epigenetic silencing of the intronic microRNA hsa-miR-342 and its host gene EVL in colorectal cancer.* (1476-5594 (Electronic)).

38. Meng, F., et al., *Epigenetic regulation of microRNA-370 by interleukin-6 in malignant human cholangiocytes.* (1476-5594 (Electronic)).

39. Saito, Y., et al., *Chromatin remodeling at Alu repeats by epigenetic treatment activates silenced microRNA-512-5p with downregulation of Mcl-1 in human gastric cancer cells.* (1476-5594 (Electronic)).

40. Brueckner, B., et al., *The human let-7a-3 locus contains an epigenetically regulated microRNA gene with oncogenic function.* Cancer Res, 2007. **67**(4): p. 1419-23.

41. Lu, L., et al., *Hypermethylation of let-7a-3 in epithelial ovarian cancer is associated with low insulin-like growth factor-II expression and favorable prognosis.* Cancer Res, 2007. **67**(21): p. 10117-22.
